# App-based serious gaming for training of chest tube insertion: study protocol for a randomized controlled trial

**DOI:** 10.1186/s13063-017-1799-5

**Published:** 2017-02-06

**Authors:** Mirco Friedrich, Christian Bergdolt, Patrick Haubruck, Thomas Bruckner, Karl-Friedrich Kowalewski, Beat Peter Müller-Stich, Michael C. Tanner, Felix Nickel

**Affiliations:** 1 Department of General, Visceral, and Transplantation Surgery, University of Heidelberg, Im Neuenheimer Feld 110, Heidelberg, 69120 Germany; 2 Center for Orthopedics, Trauma Surgery and Spinal Cord Injury, Trauma and Reconstructive Surgery, HTRG - Heidelberg Trauma Research Group, University of Heidelberg, Schlierbacher Landstrasse 200a, Heidelberg, 69118 Germany; 3 Institute for Medical Biometry and Informatics, University of Heidelberg, Im Neuenheimer Feld 305, Heidelberg, 69120 Germany

**Keywords:** Chest tube insertion, Education, Training, Serious gaming, Hematothorax, Pneumothorax

## Abstract

**Background:**

Chest tube insertion is a standard intervention for management of various injuries of the thorax. Quick and accurate execution facilitates efficient therapy without further complications. Here, we propose a new training concept comprised of e-learning elements as well as continuous rating using an objective structured assessment of technical skills (OSATS) tool. The study protocol is presented for a randomized trial to evaluate e-learning with app-based serious gaming for chest drain insertion.

**Methods:**

The proposed randomized trial will be carried out at the Department of Orthopedics and Traumatology at Heidelberg University in the context of regular curricular teaching for medical students (*n* = 90, 3rd to 6th year). The intervention group will use e-learning with the serious gaming app Touch Surgery (TM) for chest drain insertion, whereas the control group uses serious gaming for an unrelated procedure. Primary endpoint is operative performance of chest drain insertion in a porcine cadaveric model according to OSATS.

**Discussion:**

The randomized trial will help determine the value of e-learning with the serious gaming app Touch Surgery (TM) for chest drain insertion by using the OSATS score. The study will improve surgical training for trauma situations.

**Trial registration:**

Trial Registration Number, DRKS00009994. Registered on 27 May 2016.

**Electronic supplementary material:**

The online version of this article (doi:10.1186/s13063-017-1799-5) contains supplementary material, which is available to authorized users.

## Background

Chest tube insertion is an established minimally invasive surgical intervention in treatment of acute trauma patients bearing injuries of the lungs and thorax. It is the gold standard with regards to emergency care of pneumothorax or serial rib fractures as well as lesions of pulmonary vessels resulting in a hematothorax [1]. Chest tube insertion is equally performed in preclinical care and the clinical setting, yielding the need for procedural proficiency of physicians in both the field and the in-hospital emergency setting. Anatomical knowledge of the structures involved, an accurate positioning and standardized execution are crucial to a treatment, which is as quick and accurate as possible. Correct chest tube insertion facilitates efficient therapy without time loss or further complications that are potentially lethal because of the intervention’s vicinity to vital organs [2]. Thus, there is a need for a comprehensive training and assessment prior to the emergency situation that enables both versed physicians and novices to acquire proficiency in chest tube insertion. Early and decisive training using a tool as such might standardize handling of these emergency situations and could translate to improved trauma care and outcome. E-learning has been shown to be a valuable asset to surgical training [3–5]. Since computer games are believed to be commonplace for today’s students and their experience potentially enables them to profit from different approaches, the concept of ‘serious gaming’ is subject to training research [6–9]. Since serious games have already been evaluated in various situations within the framework of surgery, our aim is to expand the investigation of this multimedia training approach to trauma medicine and standard emergency interventions.

To advance and standardize training success without straining staff and resources, we use an objective structured assessment of technical skill (OSATS) for chest tube insertion to standardize expert rating [10]. The OSATS tool for chest drain insertion was developed based on key steps of correct chest tube insertion, originally published by Hutton et al., which were modified and amended by a team of trauma and general surgeons [11]. Ten representative procedural steps can be evaluated separately using a 5-point Likert scale complemented by detailed formulation of performance levels. Trainee’s performance in sub-steps adds up to a maximum total score of 50 points and a minimum score of 10 points, respectively.

The OSATS is used in a randomized controlled trial (RCT) investigating a serious gaming approach for training of chest tube insertion for medical students in a standardized and structured setting with the validated surgical training app Touch Surgery™ (TS) (Kinosis Ltd., London, UK) [12]. The study protocol for the RCT is described in the present manuscript.

## Methods and Design

### Objectives

Primary objective of this study is to evaluate training outcome of a structured chest drain training curriculum that comprises elements of serious gaming. Both operative and time-dependent performances of participants are analyzed. Secondary goals include character profiling concerning previous experience and specific extracurricular activities. Thus, participants particularly benefiting from the presented training concept as well as obstacles and positive impulses when training with standardized E-learning platforms might be identified. Evaluation of serious gaming elements in student teaching is also performed to evaluate the potential establishment of such modalities in medical school curricula.

### Study design

This is a registered prospective, single-center, rater-blinded, two-arm, parallel-group randomized controlled trial (DRKS00009994).

### Setting and participants

This study is carried out in the training center of the Department of Orthopedics and Traumatology at Heidelberg University. This study is conducted in the context of regular curricular teaching for medical students during their clinical years (3rd to 6th year) at Heidelberg University.

### Inclusion and exclusion criteria

The inclusion criterion mandates that participants are medical students in their clinical years (3rd–6th year) at Heidelberg University who have completed their 18th year of age. Previous participation in surgical training courses including insertion of chest drains is not an exclusion criterion but will be recorded and analyzed.

### Randomization

Blocked randomization, stratified by gender, is used to randomly assign participants to each group (1:1 ratio), resulting in one intervention and one control group. Randomization is performed by an employee not involved in training, rating or data collection regarding the present study using opaque, sealed envelopes. As student recruitment to the study will be completed before randomization, any influence of randomization results or subsequent task assignments is considered minimal.

### Training curriculum

**Fig. 1:**
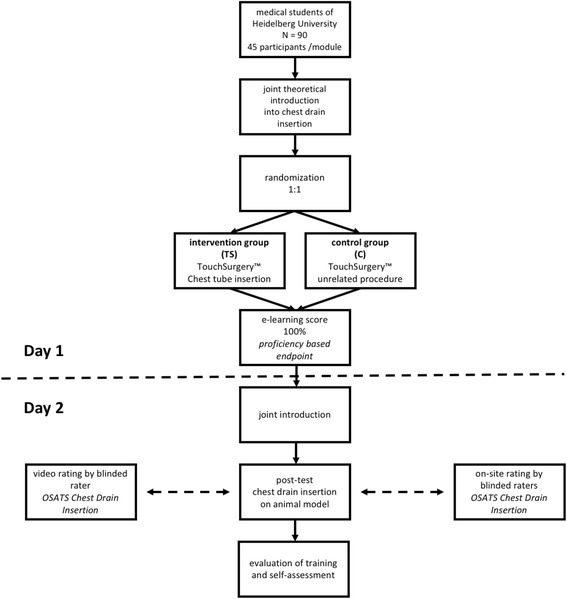
Study flow chart

Training is conducted according to the structured training curriculum outlined in Fig. 1 (study protocol flow chart) and supervised by experienced surgeons not involved in the randomization or analysis of study results. Students participate in two training sessions lasting 120 min each. At the beginning of the first session and after a theoretic introduction to the procedure, participants in groups 1 and 2 are asked to study the E-learning material assigned to them until they reach an overall app-based score of 100% on performance of the assigned module. Intervention group students use the TS module “Chest Drain Insertion,” while participants in the control group are asked to practice the unrelated module “Central Venous Catheter Insertion.” Control group participants otherwise attend every introduction and assessment included in the training sequence. Performance of the assigned training modules will be monitored to ensure adherence to each group-specific protocol. As this study is conducted in the context of the regular curriculum, dropouts, if any, are expected to be very few. Additionally, we created a highly efficient curriculum. This results in a short intervention period facilitating reduction of attrition bias as suggested by Little et al. [13].

### Assessment

Participants perform chest drain insertion on an accordingly prepared porcine model during the second training session. Performance is videotaped and rated by two different raters on-site using the modified OSATS tool for chest drain insertion (Table 1). The raters are blinded to the training status of the participants. An additional video-based evaluation is performed by a blinded rater according to the same scoring tool. The modified OSATS for chest drain insertion was evaluated in a pilot study and showed good construct validity for distinction between experience levels. Unblinding of raters or employees involved in data analysis and interpretation is not intended. To prevent selection bias, baseline characteristics including age, year of studies, previous experience, gender and hobbies will be compared. Baseline testing on a porcine model is not performed as the hypothesis is that the proposed curriculum, consisting of multimedia training material only, will directly improve performance. The aim of the training curriculum and study is to prepare novices for their first performance of chest drain placement. Baseline testing on a real model could disguise the effect of the training curriculum.

**Table 1:** Objective structured assessment of the technical skills score for chest tube insertion

Correct identification of incision location	1 **Poor** The chosen dissection plane deviates tremendously from the suggested site	2	3 **Sufficient** The chosen dissection plane deviates slightly from the suggested site	4	5 **Excellent** 4th/5th intercostal space; mid/anterior axillary line
Correct plane of dissection subcutaneously	1 **Poor** Both distance or execution of tunneling lack accuracy	2	3 **Sufficient** Either distance or execution of tunneling lack accuracy	4	5 **Excellent** Both distance and execution of tunneling are accurate
Blunt dissection on top side of rib	1 **Poor** Flawed dissection; not carried out on top side of rib	2	3 **Sufficient** Solid dissection carried out with minor errors	4	5 **Excellent** Confident cut through the subcutaneous layers and intercostal muscles
Scissors/clamp guarded with other hand during dissection and pulled out without closing the instrument	1 **Poor** Hazardous handling that might affect the patient	2	3 **Sufficient** Improvable handling	4	5 **Excellent** Confident handling of the used instruments
Digital exploration of pleural cavity on chest wall to rule out adhesions	1 **Poor** No digital exploration	2	3 **Sufficient** Finger inserted in pleural cavity	4	5 **Excellent** Digital exploration in 360° with turning of the wrist rules out adhesions
Drain guarded with hand while being inserted	1 **Poor** Hazardous handling that might affect the patient	2	3 **Sufficient** Improvable handling	4	5 **Excellent** Confident handling of the used instruments
Drain inserted into pleural cavity	1 **Poor** Tube advancement is carried out poorly	2	3 **Sufficient** Tube advancement is carried out with minor errors	4	5 **Excellent** Forceps unclamped in time and tube manually advanced.
Estimate made of drain length	1 **Poor** Estimate deviates tremendously from rater’s opinion	2	3 **Sufficient** Estimate deviates slightly from rater’s opinion	4	5 **Excellent** Optimal estimate stated
Economy of time and motion	1 **Poor** Many unnecessary/disorganized movements	2	3 **Sufficient** Organized time/motion, some unnecessary movement	4	5 **Excellent** Maximum economy of movement and efficiency
Amount of help/assistance needed from tutor	1 **Poor** Task could not be carried out without extensive assistance	2	3 **Sufficient** Trainee only raises important questions in order to maximize performance	4	5 **Excellent** Almost no assistance needed; task is carried out confidently

### Primary outcome measure

Primary endpoint is operative performance chest drain placement according to the standardized and evaluated OSATS scoring tool as measured by direct observation by the blinded raters during the course. Gaussian distribution of the total score can be assumed.

### Secondary endpoints

Operative performance by gender- and experience-dependent subgroup analyses, time-dependent performance, correlation with extracurricular activities and hobbies, subjective self-assessment as well as possible differences and bias between on-site and video ratings are examined as secondary endpoints [14].

### Statistical analysis

Mean and standard deviation in case of continuous data, with absolute and relative frequencies for categorical parameters, are used to describe the distributions of all parameters of interest. Possible differences of the primary outcome and OSATS score will be tested using analysis of covariance with intervention group as factor, participants’ self-attributed expertise score as covariate and OSATS score after intervention as dependent variable. Secondary endpoints will be descriptively analyzed according to their respective distribution with t-tests in case of continuous data and chi-square test in case of categorical data. Multiple imputation will be applied to compensate for any missing data. If it is found to be appropriate, graphical statistical methods will be deployed to illustrate findings.

### Sample size determination

According to results of the pilot study with an identical primary endpoint and conducted in a similar context, group size was determined to be 45 participants randomized to each group. The OSATS for chest drain insertion evaluated in the pilot study will be used as primary endpoint with a relevant mean difference of 3 ± 5 points in the scoring system. Differences can be detected with a significance level α = 0.05 and a power of 1-β = 0.8. Intervention group sizes will exceed this determined sample size.

## Discussion

This study aims to teach important aspects of trauma surgery to medical students using elements of serious gaming. We believe this approach proves additionally beneficial to outcome when teaching today’s digitally experienced medical trainees. Our hypothesis is both that participants will profit from the supplemented training curriculum and that the modified OSATS tool can subsequently objectify their improved skill level. This study will therefore help to investigate potential improvements of current curricula by serious gaming and may even provide a reliable training control for medical trainees with the modified OSATS aiming at a safe execution of this crucial emergency procedure. Serious gaming might as a result be integrated into modern medical school curricula for surgical residents as well. Further investigations and outcomes of this study will increase the available knowledge about criteria to be met in order to ensure optimal surgical training for trauma situations.

### Trial status

The randomized controlled trial recruitment and conduction of assessments are planned from June to December 2016. Data analysis and evaluation will be performed subsequently. Final results of this study will be published.

## Additional file

Additional file 1: SPIRIT checklist for interventional trials. (DOC 124 kb)
